# Near-infrared spectroscopy discriminates mass-reared sterile and wild tsetse flies

**DOI:** 10.1371/journal.pntd.0012857

**Published:** 2025-01-29

**Authors:** Soumaïla Pagabeleguem, Dari F. Da, Bernard M. Somé, Marx S. P. Avelessi, Nicaise D. C. Djègbè, Rebecca L. Yoda, Abdramane Bagayogo, Hamidou Maïga, Thomas S. Churcher, Roch K. Dabiré

**Affiliations:** 1 Institut des Sciences de l’Environnement et du Développement Rural, Université de Dédougou (UDDG), Dédougou, Burkina Faso; 2 Direction Génnérale de l’Entomologie et de la Lutte contre les Maladies Animales à vecteurs (DGELMA), Bobo-Dioulasso, Burkina Faso; 3 Institut de Recherche en Sciences de la Santé, Direction Régionale, Bobo-Dioulasso, Burkina Faso; 4 MRC Centre for Global Infectious Disease Analysis, Department of Infectious Disease Epidemiology, Imperial College London, London, United Kingdom; International Atomic Energy Agency, AUSTRIA

## Abstract

**Background:**

Monitoring the efficacy of the sterile insect technique (SIT) programs, it is desirable to discriminate between wild and sterile tsetse males captured in monitoring traps. Currently, this is primarily achieved by marking sterile males with fluorescent dye powder before release, and identifying them using a fluorescence camera and/or microscope. However, the accuracy of this method is limited due to defective marking and wild flies contaminated with a few dye particles in the monitoring traps. Molecular techniques have been developed to discriminate doubtful flies, but they are expensive for endemic countries.

**Methodology/Principal findings:**

Here, we investigate the ability of a new generation monitoring tool, Near-Infrared Spectroscopy (NIRS), to discriminate between laboratory-reared *Glossina palpalis gambiensis* males and their field counterparts. NIRS was able to discriminate wild males from laboratory-reared males with 86% accuracy. Notably, the prediction accuracy improved to 88% when the laboratory-reared flies had been irradiated.

**Conclusions/Significance:**

These findings suggest that NIRS can successfully identify tsetse flies even when UV camera identification is inconclusive. However, further studies are needed to expand the training dataset and include additional environmental variables before validating NIRS as a complementary method for future tsetse eradication programs.

## Author summary

In the context of area-wide integrated pest management (AW-IPM) approaches including a sterile insect technique (SIT), to monitor the efficacy of the program, it is desirable to discriminate between wild and sterile males captured in monitoring traps. Currently, this is primarily achieved by marking sterile males with fluorescent dye powder before release, and identifying them using a fluorescence camera and/or microscope. However, the accuracy of this method is limited due to defective marking and wild flies contaminated with a few dye particles in the monitoring traps. The molecular tool developed to discriminate doubtful flies remains expensive for endemic countries. We investigate the ability of the Near-Infrared Spectroscopy (NIRS) to discriminate between reared *Glossina palpalis gambiensis* males and their field counterparts. NIRS discriminated wild males from reared males with high accuracy and can successfully identify tsetse flies even when UV camera identification is inconclusive. This tool has the potential to be a complementary method for future tsetse eradication programs with a SIT component.

## Introduction

Tsetse flies (*Glossina spp*.) are the only cyclic vectors of trypanosomes, responsible for sleeping sickness or Human African Trypanosomosis (HAT) in humans and nagana or African Animal Trypanosomosis (AAT) in livestock across sub-Saharan Africa [1]. Tsetse flies infest 38 countries in the region, severely limiting the development of sustainable and productive agricultural systems over more than 10 million km^2^ [2]. Over 60 million people are at continuous risk of trypanosomosis infection, and farmers in tsetse-infested areas experience losses of 20–40% in livestock productivity leading to an estimated annual economic loss of approximately USD 4.75 billion [3].

Four methods are considered both environmentally and economically acceptable to eliminate or eradicate these vector-borne diseases, which can be used in the context of area-wide integrated pest management (AW-IPM) approaches. These strategies include the sequential aerosol technique, the use of insecticide-impregnated traps/targets, Life bate technologies utilizing pour-on, and the sterile insect technique (SIT) [1]. AW-IPM programs with an SIT component has been successfully used throughout the world to suppress, eradicate, contain or prevent the introduction of several insect pests such as fruit flies [4], moths [5], screwworm [6], mosquitoes [7] and tsetse flies [8]. Furthermore, the effectiveness of SIT has been demonstrated in the eradication of tsetse populations across several African countries such as Zanzibar, Nigeria, and Burkina Faso where different species being targeted [8–11]. In Senegal, an ongoing program aims to eradicate a population of *G*. *palpalis gambiensis* from the Niayes area [12–14].

In AW-IPM programs with an SIT component, the status of the field-trapped flies (whether mass-reared sterile males or wild fertile males) remains one of the most crucial prerequisites for success. Indeed, the ratio of sterile to wild flies is an important parameter in monitoring the efficiency of a SIT campaign [8,15,16]. The discrimination between sterile and wild males relies mainly on the fluorescence marking of insectary-reared flies. The marking process is done during the adult’s emergence using a fluorescent dye powder (DayGlo) mixed with sand or after emergence by impregnation with the powder. Emerging male flies would pick up the powder whilst crawling through the sand, especially in the ptilinum that would later be retracted into the head capsule [17]. This treatment discriminates sterile from wild flies using a fluorescence camera and /or a fluorescence microscope [12]. However, marking sterile males with fluorescent dye-powder presents several limitations. Indeed, the fluorescent dye powder is not infallible, and some sterile flies may be poorly marked with only a few particles of dye. Conversely, wild flies may become contaminated in the monitoring traps with some powder particles by coming into contact with their sterile counterparts, leading to confusion. Additionally, flies may also be predated by ants in the traps and then lose their head, which makes it difficult to accurately determine their origin. To address these issues, a molecular tool has been developed allowing discrimination with high accuracy between the mass-reared sterile and wild males [17]. While highly accurate, this method is a bit expensive and time-consuming, especially when analyzing a large number of sample results [17]. Alternative tools are therefore urgently needed that can accurately, rapidly, and cost-effectively discriminate sterile and wild tsetse males.

The Near-infrared spectroscopy (NIRS) technique, a promising new-generation monitoring tool, has shown potential as a reliable alternative for identifying tsetse flies. NIRS operates by measuring the absorption of near-infrared energy by biological samples at specific wavelengths. Near-infrared spectroscopy relies on the direct relationship between the physical and chemical properties of a substance and its absorption at particular wavelengths within the near-infrared region of the electromagnetic spectrum. Near-Infrared Spectroscopy (NIRS) focuses on organic molecules composed of carbon, oxygen, hydrogen, and nitrogen atoms connected by covalent bonds. These elements are also present in the Diptera cuticle, whose absorbance properties are unique to each species. This distinct composition can be utilized in models for classifying mosquito species [18]. When Diptera are examined, near-infrared radiation reveals information about them by analyzing the stretching and bending of C-H, O-H, and N-H bonds within their cuticles. Furthermore, during scanning, the reflectance (R) of radiation at each wavelength is measured by detectors and converted into absorbance values (Log1/R). It is rapid, non-destructive, cost-effective, reagent-free method that requires minimal technical training once algorithms have been developed [18–20]. NIRS has been applied in malaria research to predict mosquito age, identify *Anopheles* species and determine *Plasmodium* infection status [18,21–23]. It has also been used to differentiate triatomine species [24] and *Biomphalaria* species [25], as well as to separate tsetse pupae by sex [26]. NIRS has also been explored for detecting *Trypanosoma cruzi* in midgut, rectum and excreta samples of *Triatoma infestans* [27]. This study aims to determine whether the NIRS technique can accurately distinguish between unmarked wild-caught and laboratory-reared, sterile and marked male *Glossina palpalis gambiensis*. Additionally, it investigates whether irradiation or the marking processes in the laboratory influence the accuracy of NIRS predictions.

## Methods

### Insectary flies

The *Glossina palpalis gambiensis* flies used in the experiments came from a colony maintained at the Insectarium de Bobo-Dioulasso (IBD) of the Direction Générale de l’Entomologie et de la Lutte contre les Maladies Animales à vecteurs (DGELMA) under standard conditions (25 ± 1 °C, 75 ± 5% relative humidity and 12:12 light:dark photoperiod). Historically, the colony was derived from a strain established in 1972 using wild flies collected in Guinguette, a locality near to Bobo-Dioulasso, Burkina Faso [28]. This resulting colony was mass-reared at the “Centre International de Recherche-Développement sur l’Elevage en zone Subhumide (CIRDES)” [29]. In 2016, approximately 54,000 adult flies from the CIRDES colony were transferred to the IBD insectary, serving as the foundation for a new mass-rearing colony [30].

Three experimental groups of insectary-reared *G*. *palpalis gambiensis* flies were set up: unmarked fertile, unmarked sterile and marked sterile flies. The unmarked fertile flies were sampled from newly emerged colony flies, divided into Roubaud cages (20 flies per cage 4.5 cm × 13 cm × 8 cm), and kept under standard conditions. For the sterile flies, batches of pupae from which almost all females have emerged (approximately three days after the onset of emergence) were chilled at 8°C and irradiated with 120 Gy using a Cobalt 60 source (Foss Therapy Services, Inc. model 812 S/N 002) [31]. To obtain marked sterile flies [17], irradiated pupae were placed in Petri dishes under ~1 cm of sand mixed with a fluorescent dye (DayGlo) (0.5 g dye / 200 g of sand) to mimic natural emergence conditions and marking the flies during emergence [32]. This marking process is essential for distinguishing sterile males from wild flies during monitoring as part of SIT program progress assessment [15]. For the unmarked sterile flies, irradiated pupae were emerged without the marking device.

Flies from three experimental groups were fed with irradiated bovine blood using an *in vitro* silicon membrane system and maintained under standard insectary conditions. For the NIRS scanning, from each experimental group, an average of 30 flies were sampled at five-day intervals starting from the day of emergence, continuing until the flies were 30 days old (day 1, day 5, …., day 25 and day 30) and immediately shipped to the Institut de Recherche en Sciences de la Santé (IRSS) laboratory for analysis.

### Wild flies

Wild flies were collected from the Bama forest (11.38454N, 4.409087W), located approximately 25 kilometers from Bobo-Dioulasso. This forest, a small area (500 m long and 130 m wide), has permanent water, protected vegetation, and a variety of animals such as monitor lizards (*Varanus niloticus*), crocodiles (*Crocodylus niloticus*), domestic animals and humans that likely constituting the main feeding sources for the *G*. *palpalis gambiensis* population. During two field trips in September and October 2021, each lasting five days, tsetse flies were trapped using six biconical traps [33] and five monoconical traps [34]. The captured flies were identified morphologically by sex and species according to Pollock (1982) [35]. The flies were then placed in humidified containers and transferred to the IRSS laboratory, where they were immediately scanned. Both males and females were scanned and only males were included in analysis. The wild flies were collected from an area without an ongoing SIT program so all males were assumed to be fertile.

### Flies scanning

Flies from IBD were euthanized by exposure to chloroform vapor for three minutes. Afterward, each fly was scanned using a LabSpec4 Standard-Res i (standard resolution with an integrated light source) near-infrared spectrometer and a bifurcated reflectance probe mounted 2 mm from a spectralon white reference panel (ASD Inc., Westborough, Massachusetts, USA) [36]. All specimens were scanned centering the head and thorax region under the focus of the light probe, and the RS3 spectral acquisition software (ASD Inc., Malvern PANalytical) was used to record the flies’ spectra (S1 Fig). This software automatically records the average spectra from 20 scans. Absorbance data was recorded across a range of 350 to 2500 nm in the electromagnetic spectrum.

### Data analysis

The statistical analysis was performed based on the spectra obtained from scanning both laboratory-reared and wild flies. Five main comparisons were undertaken: (A) unmarked sterile *versus* wild-caught flies which is the primary comparison relevant for the SIT programs as it helps distinguish between sterile males released during the program and wild tsetse flies, (B) unmarked sterile *versus* marked sterile flies to understand the ability of the NIRS technique to determine the marking status (marked/unmarked) of laboratory-reared flies, (C) unmarked sterile *versus* unmarked fertile flies to know whether the NIRS could identify the sterilization status (sterile/fertile) of laboratory-reared tsetse flies, (D) sterile-marked tsetse flies versus wild-caught to investigate whether NIRS could be used alone in the SIT programs and finally (E) unmarked fertile *versus* wild-caught flies to determine the origin status.

Machine learning methods were used to construct binomial logistic regression models using maximum likelihood estimation. For each tsetse fly, the mean of the two scans was used for the analysis. The spectra data were then trimmed to values corresponding 500 and 2350 nm to eliminate the excess noise arising from the sensitivity of the spectrometer at the end of the near-infrared range. These spectra were analyzed using partial least squares regression (PLS), a statistical technique utilizing the covariance between the spectra and marking status in order to extract the most informative elements within a much smaller dimension. All analyses used simple models that did not include spectra smoothing or penalized coefficient function estimation. The data were divided into three subsets (at a 3:1:1 ratio) for training to build the model, validation to choose the optimal number of principal components (from 2 to 50), and testing to estimate the model’s generalized error. All models used random sampling to ensure an equal number of observations per class. This procedure was repeated 100 times, and the results were averaged across all realizations to improve robustness. The accuracy of the model was evaluated using the area under the receiver operating characteristic (AUC) curve, where a value closer to 1 indicates better model performance). All analyses were performed in R using the *mlevcm* package [37]. The full spectral data set and data analysis details are available in S1 Table and S1 Data, respectively.

## Results

**Fig 1:**
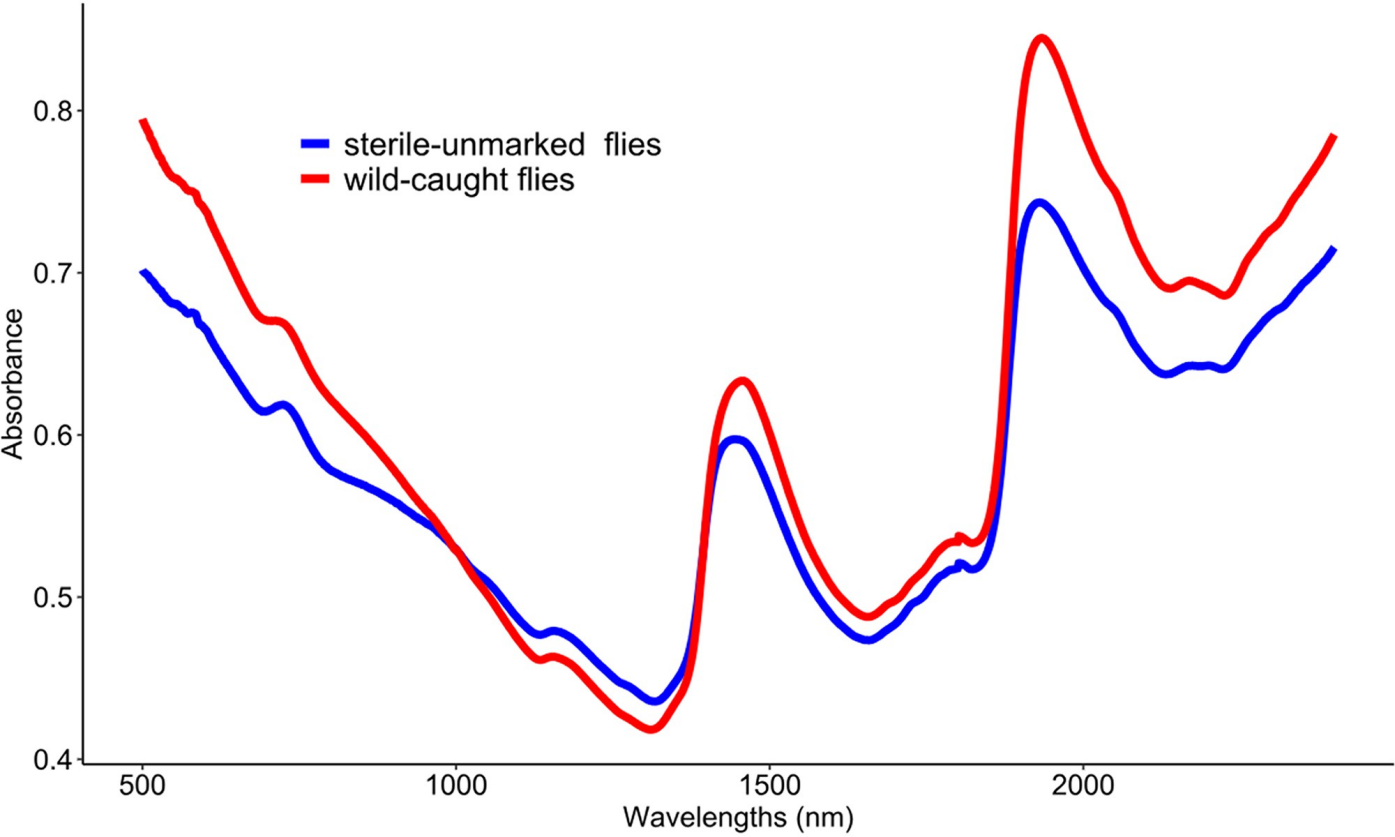
Average spectra of laboratory sterile-unmarked flies (n = 114) and wild-caught flies (n = 143), each being sampled at discrete wavelengths in the interval [500, 2400 nm].

A total of 627 laboratory-reared males of *G*. *palpalis gambiensis* aged from 1 to 30 days and 143 wild-caught males were used to determine the predictive discrimination ability of the model. The spectra profile revealed noticeable differences between certain experimental groups. Specifically, the average spectra from wild-caught flies showed higher absorbance levels compared to laboratory-reared, sterile-unmarked flies (Fig 1).

According to the methodology describe above, cross-analyses between the groups of *G*. *palpalis gambiensis* taking into account the interventions operated during fly rearing in the laboratory (marking and irradiation) and their origin were performed to understand the effect of each intervention.

**Fig 2:**
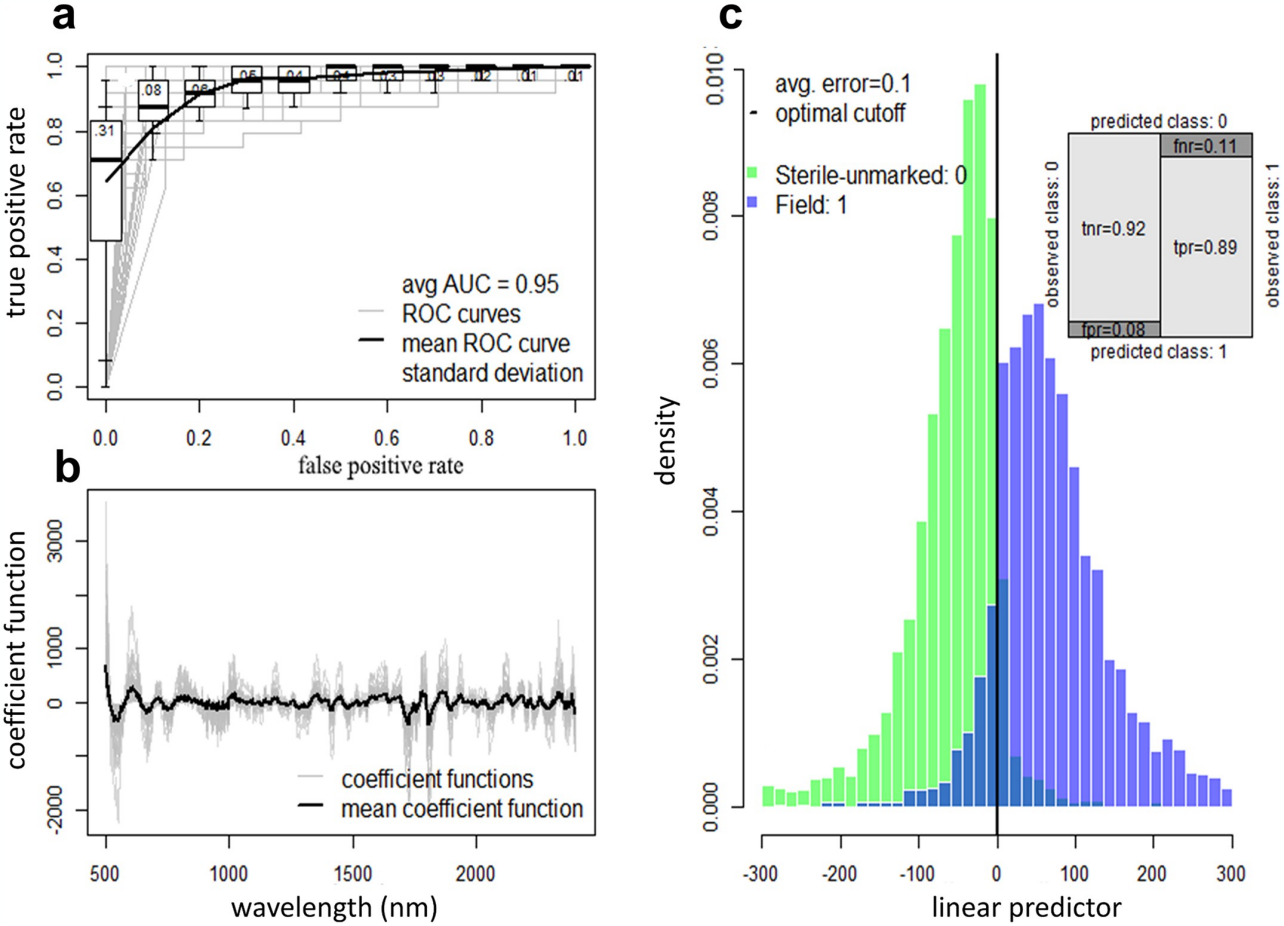
NIRS ability to discriminate between sterile unmarked and wild-caught flies. (**a**) Receiver operating characteristic (ROC) curve illustrating the diagnostic ability of the best-fit model. Overall performance is given by the average area under the ROC curve (AUC). A theoretical perfect diagnostic would be in the top left corner. The average ROC curve shown by the solid line with boxplots shows the variability for 100 randomizations of the training, validation and testing. (**b**) Coefficient functions for the best-fit model for each of the 100-dataset randomizations (grey lines) and the overall average (black line). (**c**) Histogram of the estimated linear predictor for the test observations, color-coded by the true class: marked irradiated flies (light blue colored bars) or Wild-caught flies (green bars). Vertical solid black line indicates the best threshold for differentiating marked irradiated flies and wild-caught flies. Darker blue bars indicate where the two distributions overlap and misclassified flies’ status. Misclassified wild-caught *Glossina* are shown to the left of the optimal classification threshold line and misclassified sterile unmarked *Glossina* to the right. Inset shows the confusion matrix illustrating the different error rates: true negative rate (tnr) for the sterile unmarked flies correctly classified; false negative rate (fnr) for the misclassified field flies; false positive rate (fpr) for the misclassified sterile unmarked flies; and true positive rate (tpr) corresponding to the field flies correctly classified.

Comparison A determined the ability of NIRS to differentiate between sterile unmarked and wild tsetse flies, achieving an overall accuracy of 90% [sterile-unmarked: 92%; wild-caught: 89%; (likelihood of NIRS accurately classifying each group); Fig 2].

**Fig 3:**
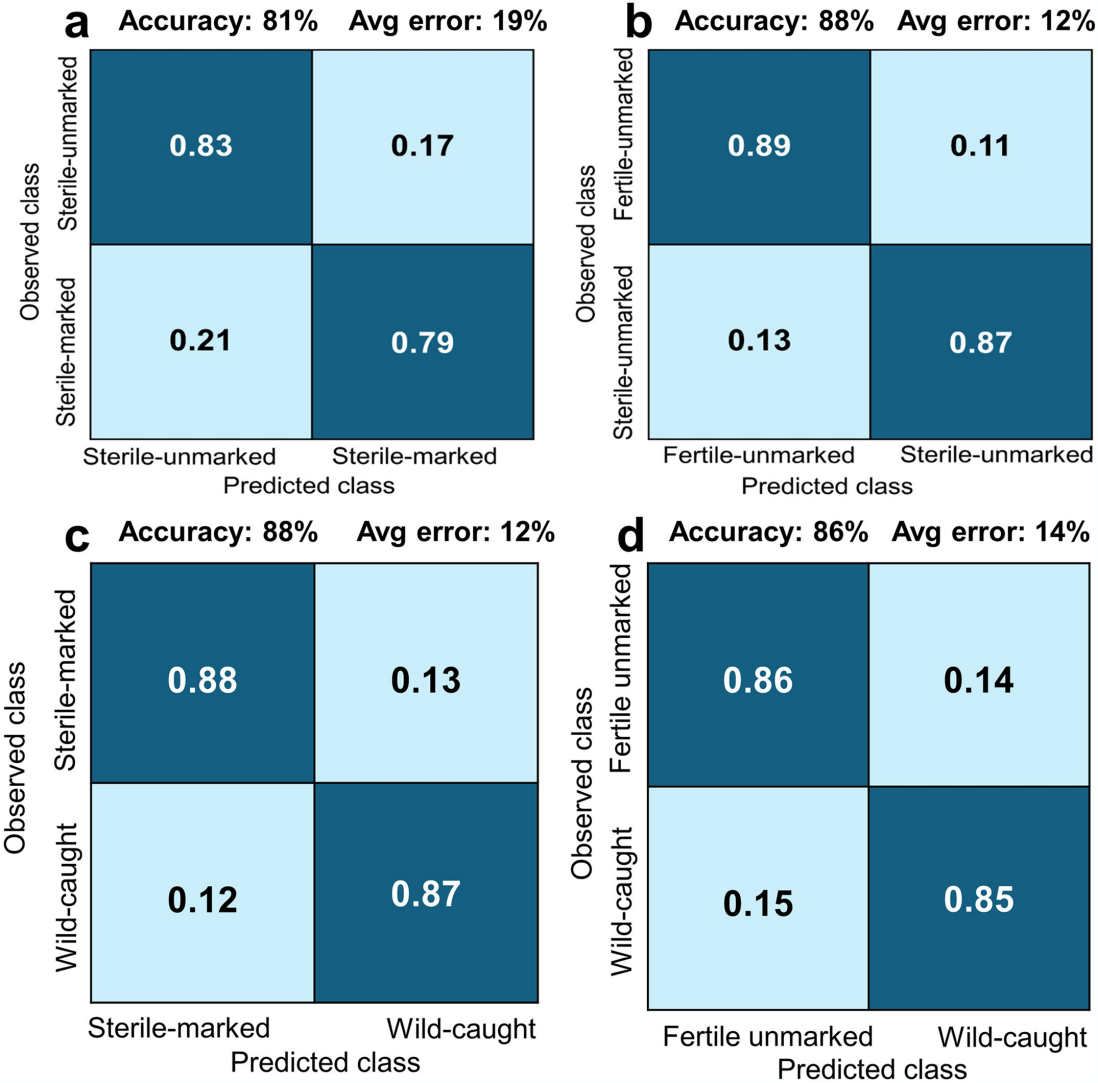
NIRS accuracy in predicting laboratory-reared and wild-caught *Glossina*. **a**: Marking effect (sterile-unmarked *vs* sterile-marked); **b**: Irradiation effect (fertile unmarked *vs* sterile unmarked); **c**: effect (sterile marked *vs* wild); **d**: Origin effect (laboratory fertile and unmarked *vs* wild).

Comparison B using the group of sterile tsetse flies demonstrated that NIRS was also able to distinguish between marked and unmarked sterile flies with an accuracy of 81% (sterile-unmarked: 83%; sterile-marked: 79%; Fig 3a). The predictive models developed using spectra collected from laboratory unmarked and fertile versus laboratory unmarked sterile flies (Comparison C) showed a similar overall accuracy of 88% (fertile-unmarked: 89%; sterile-unmarked: 87%; Fig 3b). When NIRS have been tested to classified sterile-marked tsetse flies versus wild-caught (Comparison D), the accuracy still better with 88% (sterile-marked: 88%; wild-caught: 87%; Fig 3c). Then, without any intervention operated during flies rearing in the insectary (Comparison E), NIRS successfully discriminated between unmarked fertile flies and wild-caught flies with an accuracy of 86% (fertile unmarked: 86%; wild-caught: 85%; Fig 3d).

## Discussion

The status of field-caught male tsetse (sterile or wild) is a crucial entomological parameter for area-wide programs that includes the sterile insect technique component. Current methods, such as the fluorescence marking of sterile flies, can sometimes be defective, making it challenging to distinguish between wild and sterile males using a fluorescence camera. To address these limitations, the exploration of NIRS as a monitoring tool for trypanosomosis vectors offers a promising alternative to improve the accuracy and reliability of such programs.

We showed that NIRS could be applied to accurately and rapidly discriminate between laboratory-reared sterile from the wild-caught males (Comparison A). This technique could differentiate with high accuracy (90%) the unmarked-sterile laboratory-reared flies from their wild-caught counterparts. Notably, a similar reliability was observed when the sterile males were marked with fluorescence powder (Comparison B) suggesting that marking with fluorescent powder does not appear to have any impact on the NIRS ability to discriminate between sterile and wild flies. The sterile males were not marked with fluorescent powder to better mimic the conditions of doubtful flies (having lost the fluorescent powder) and thus could not be distinguished from wild males by a fluorescence camera. Alternatively, it allows exploration of a scenario when sterile males are not marked when they are released into the field. The mechanism by which NIRS was able to differentiate between these two groups of flies remains unclear. However, two likely hypotheses could be: (i) the irradiation of laboratory-reared tsetse flies and or (ii) underlying differences in the genetic composition of the laboratory colony or their environmental living conditions. Experiments to explore the contribution of both these factors were undertaken. Nevertheless, the results were inconclusive as the accuracy of NIRS to differentiate different groups of tsetse was similar suggesting evidence for both hypotheses and that neither is more likely. Each of the different hypotheses and the evidence to support that hypothesis are discussed in turn.

Hypothesis (i): the effect of irradiation was assessed between irradiated and non-irradiated laboratory males (Comparison C) and NIRS was able to differentiate these two groups with 88% accuracy. The consequence of the irradiation on male flies does not only induce dominant lethal mutations in the gametes [38], but also causes somatic damage in the bodies of flies which leads to a reduction of the average life-span of irradiated males as compared with untreated ones [39,40]. These processes of deterioration of cellular contents caused by irradiation in flies could play a part in the differentiation between sterile laboratory flies and their wild counterparts by the NIRS. Based on these results, the Glossina irradiation during the rearing (even without marking) could contribute to improving the NIRS ability to discriminate between sterile flies and wild-caught flies. This is interesting because the NIRS could be used as a complementary tool to discriminate doubtful flies (which have lost their marking) from the fluorescent camera.
Hypothesis (ii): the impact of colony tsetse was assessed looking at the difference between laboratory-reared fertile males and wild-caught males (Comparison E) and had an accuracy of 86%. These findings suggest that laboratory-reared flies exhibit natural differences from their wild counterparts, which NIRS can accurately detect. This difference could be due to environmental impacts such as rearing temperature, humidity, nutriment and microbiome, amongst others, which could impact tsetse fly structure and metabolism. Indeed, while laboratory flies are reared under controlled environmental conditions for over half a century, wild flies live in more natural setting conditions with important variations of multiple variables not measured here. This could lead to differences in body cellular composition between the two groups of flies and contribute to this discrimination. In addition, it has been shown that maintaining the same *G*. *palpalis gambiensis* colony under artificial conditions for 43 years led to selection pressures which resulted in differences in sequences of the Cytochrome Oxydase I gene between the laboratory flies and their wild counterparts [17]. It is also possible that the age structure of laboratory and wild flies is different (as the age of wild-caught flies is unknown) and NIRS has been shown to be able to detect differences in age [21]. Nevertheless, the high predictive accuracy of Comparison E suggests that this is not likely to be the primary cause of the differences between the samples as many flies in the two groups will be of a similar age.

This set of experiments compared wild and laboratory-reared flies and further work is needed to verify the difference between wild and laboratory reared flies that have been released and recaptured. This experiment would require molecular identification of the different groups of flies and will be essential to verify the use of the technique for monitoring SIT programs. These flies will have experienced more similar environment conditions as adults, though the comparison of fertile and infertile laboratory flies, which were otherwise identical, suggests that this is unlikely to substantially diminish accuracy. It is unsurprising that NIRS was able to differentiate marked and unmarked flies (Comparison B), though accuracy was no better than other comparisons. The machine learning methods used to differentiate between groups of spectral are improved by increasing the number of samples in the training dataset. It is therefore likely that the accuracy of the method could increase as more flies are added to the analysis. Further work is needed to build datasets with a greater range of covariates, which reflect the diversity of tsetse flies observed in the wild. For example, field samples from a greater geographic range will be needed to explore the reliability of the method across programmatically important regions with different environmental conditions.

In summary, the NIRS technique has shown strong potential in differentiating key groups of Glossina with high accuracy, making it a promising complementary monitoring tool in trypanosomosis control programs. A molecular tool based on the mitochondrial COI (cytochrome oxidase I) gene has been developed and allowed to differentiate accurately wild tsetse males from sterile ones with high accuracy [17]. However, while this method is accurate, it is a bit expensive, time-consuming for a large-scale sample analysis, destructive, laborious and requires well-trained personnel, therefore limiting its application in evaluating AW-IPM programs with a SIT component. In contrast, the NIRS technique offers a more efficient and cost-effective solution for identifying a larger number of trapped tsetse flies. Although the initial cost of the instrument is approximately 40,000 USD, ongoing operational costs are minimal. The system is field-portable, battery-powered, and requires only basic training in computer operation after calibrations have been set. Additionally, NIRS becomes increasingly cost-effective as the number of samples increases [18]. Another benefit of NIRS is that samples can be stored for at least two weeks and then scanned with high accuracy [41,42].

The NIRS technique could be an important complementary tool when discrimination between sterile and wild tsetse flies with the fluorescence camera is doubtful. To the best of our knowledge, this is the first study to report the utility of the NIRS technique to accurately and rapidly determine the status (sterile or wild) or the origin (laboratory/field) of tsetse flies independently of the marking criteria. However, an error rate of 14–10% obtained with the NIRS is not acceptable in SIT programs, leaving no space for doubtful identification, because according to the status of the flies capture in monitoring traps, decisions will be taken to continue or stop the eradication process and it could cost or save millions dollars. Based on these encouraging results, the NIRS technique could be optimized through further investigations to promote this new generation monitoring tool. The tsetse eradication program currently underway in the Niayes of Senegal [13] would be an opportunity to test this new method for validation.

## Conclusion

Near-infrared spectroscopy has the potential to be an effective entomological surveillance tool for discriminating unmarked-sterile tsetse males from their wild counterparts. This study is the first assessing the ability of the NIRS technique to discriminate between tsetse flies with high accuracy. Despite these promising results, future research should focus on integrating environmental variables into spectral analysis to improve the NIRS accuracy in identifying and monitoring tsetse flies.

## Supporting information

S1 Table Database for fly analysis. (CSV)

S1 Fig Tsetse fly spectra collecting. (TIF)

S1 Data Data analysis details. (PDF)
