# Supplementary figures and images for: Near-infrared spectroscopy discriminates mass-reared sterile and wild tsetse flies
Source: PLoS Negl Trop Dis. 2025 Jan 29;19(1):e0012857. doi: 10.1371/journal.pntd.0012857 (PMC11809883; doi:10.1371/journal.pntd.0012857)

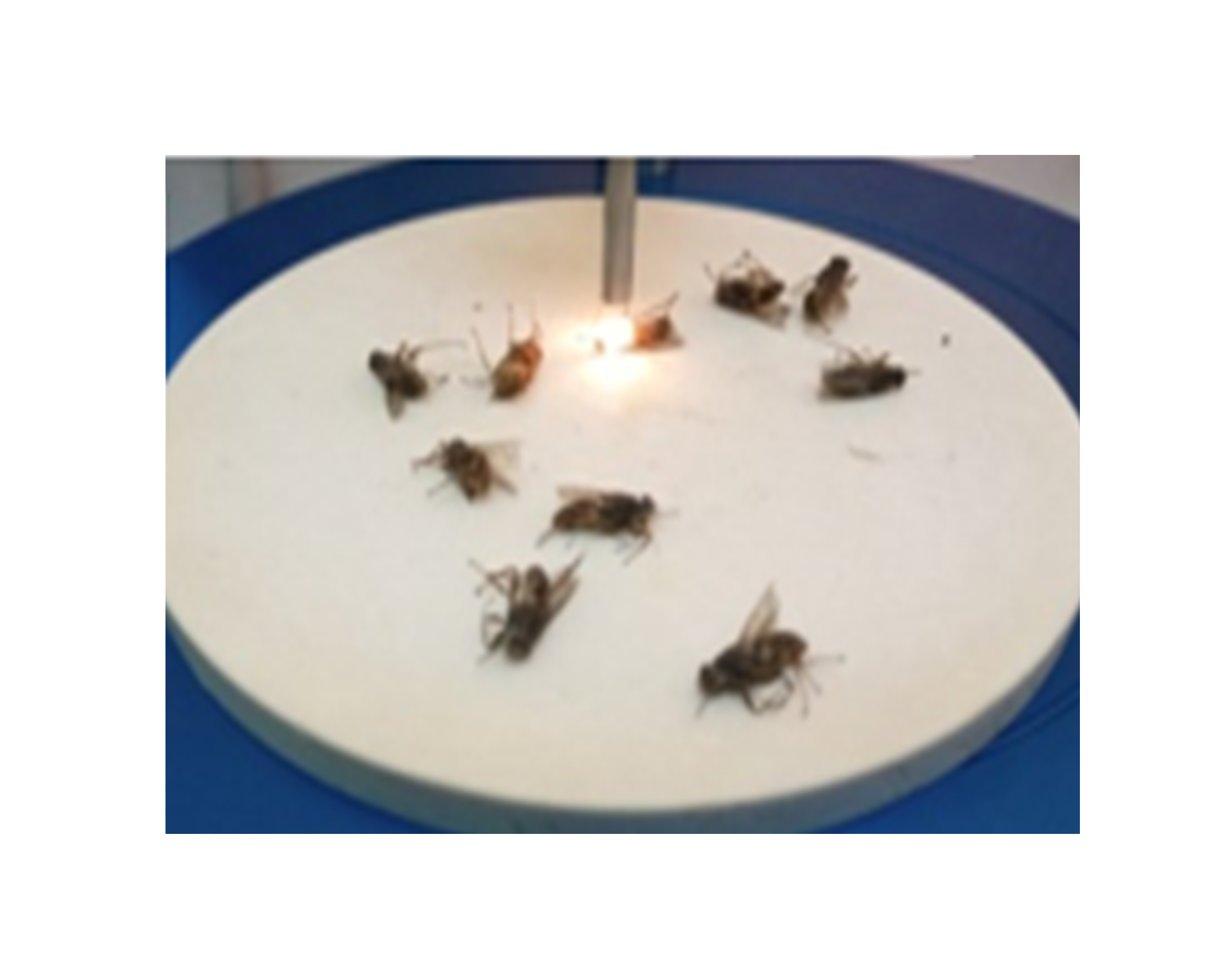

Supplement: S1 Fig — (TIF) [file pntd.0012857.s002.tif]
