# Supplementary material for: Near-infrared spectroscopy discriminates mass-reared sterile and wild tsetse flies
Source: PLoS Negl Trop Dis. 2025 Jan 29;19(1):e0012857. doi: 10.1371/journal.pntd.0012857 (PMC11809883; doi:10.1371/journal.pntd.0012857)
Supplement: S1 Data — (PDF) [file pntd.0012857.s003.pdf]

```
#Analyses de la capacité du NIRS à déterminer le statut de markage et
#d'irradiation des glossines males(Lab et sauvages).
#############################"Field"Versus"irradiated-unmarked"####################
rm(list = ls())
require(devtools)
setwd("C:/Users/Mouonniba B. SOME/Downloads/mlevcm")
#install.packages(c("rlang","caret", "dummies", "fda", "glmnet", "gplots", "penalized", "pls", "ROCR"))
#install.packages("mlevcm-master", type = "source", repos = NULL)
require("mlevcm")
################################################
data<- read.csv("C:/Users/Mouonniba B. SOME/Desktop/Master Marx/Complement d'analyse_2024/Glossina Analysis_07052024.csv", header=T,sep = ";", na.strings = "NA")
orig.id<-data[,"Glossina.ID1"]
data<-cbind(data,"Glossina.ID1")
target_marked = c("irradiated-unmarked","Field")


# read data
select <- (!is.na(data$Marking)) & (data[,"Marking"] %in% target_marked)#(!is.na(data$Age))
str(data)
unique_IDs <- unique(data[select,"Glossina.ID1"])
##following line removes all lines that are important which we have NAs for and can specifiy if you want a selection of rows (i.e. generation)
idx <- list()
for(i in unique_IDs){
  rng <- which(data[,"Glossina.ID1"] == i)
  aux <-  (!is.na(data[rng,"Marking"])) & (data[,"Marking"] %in% target_marked)# (!is.na(data[rng,"Age"]))
  idx[[i]] <- rng[aux]
}
n_replics <- unlist(lapply(idx, length))  # store how many repeated observations for each specimen
```

```
##########################################

# read predictor (X)

dates<-        c("24August_21", "25August_21","29August_21","03Sept_21","05Sept_21","08Sept_21","10Sept_21","13Sept_21","14Sept_21", "15Sept_21","16Sept_21","17Sept_21","18Sept_21","19Sept_21", "21Sept_21","23Sept_21","24Sept_21","27Sept_21","28Sept_21","30Sept_21","02Oct_21","03Oct_21","05Oct_21","06Oct_21","08Oct_21","11Oct_21","13Oct_21","18Oct_21","19Oct_21","20Oct_21","21Oct_21","22Oct_21", "23Oct_21","28Oct_21", "20Feb_23","23Feb_23","26Feb_23", "01Mars_23","04Mars_23" )

nx_dates_start <- c(  0 ,        79,       121,       223,       403,       435,     707,       739,    959,            1029 ,     1043,      1221,      1393,      1585,      1695,      1753,      1929,    2001,      2041,      2215,      2283,     2337,     2555,      2593,     2625,     2759,      2789,    2893,    2975,      3213,     3299,      3407,     3499,     3595,  6075,   7067,      7161,       7253,    7343)

nx_dates_end   <- c(  78,         120,       222,       402,       434,      706,       738,        958,    1028,            1042,      1220,      1392,      1584,      1694,      1752,       1928,      2000,    2040,       2214,      2282,      2336,     2554,      2592,      2624,      2758,    2788,      2892,    2974,    3212,       3298,     3406,     3498,       3594,   3644,  6166,  7160,      7252,    7342,      7428 )

nx_dates <- nx_dates_end - nx_dates_start + 1

nx <- sum(nx_dates)# number of spectra files

dx <- c()

for(day in dates){

  dx_aux <- matrix(0, nx_dates[which(day==dates)], 2151);  kk <- 1

  for(k in nx_dates_start[which(day==dates)]:nx_dates_end[which(day==dates)]){

    if(day %in% c("24August_21", "25August_21","29August_21","03Sept_21","05Sept_21","08Sept_21","10Sept_21","13Sept_21","14Sept_21", "15Sept_21","16Sept_21","17Sept_21","18Sept_21","19Sept_21", "21Sept_21","23Sept_21","24Sept_21","27Sept_21","28Sept_21","30Sept_21","02Oct_21","03Oct_21","05Oct_21","06Oct_21","08Oct_21","11Oct_21","13Oct_21","18Oct_21","19Oct_21","20Oct_21","21Oct_21","22Oct_21", "23Oct_21","28Oct_21")){

      f_name <- paste0("C:/Users/Mouonniba B. SOME/Desktop/Master Marx/Glossina data Age specise2/Age-specise_",day,"_", formatC(k, width = 5,flag = 0),".txt")

    }

    else{
```

```
    f_name <- paste0("C:/Users/Mouonniba B. SOME/Desktop/Master Marx/Complement d'analyse_2024/Glossina data analysis 2024/Age-specise_",day,"_", formatC(k, width = 5,flag = 0),".txt")
    }
    dx_aux[kk,] <- read.table(file = f_name, header=T, sep="\t")[,2]
    kk <- kk + 1
  }
  dx <- rbind(dx, dx_aux)
}
data_X <- dx[select,]
# read response (y)
i_y <- select #& ((1:nrow(data)) %in% unlist(sapply(idx, "[[", 1)))
data_y <- data[i_y,"Marking"]
data_z <- data[i_y,]

setwd("C:/Users/Mouonniba B. SOME/Desktop/Master Marx/Complement d'analyse_2024")
  #write your new files
  write.csv(data_X,"Glossina_marking-lab-Fiel_ x.csv",row.names=FALSE)
  write.csv(data_y,"Glossina_marking-lab-Fie_ y.csv",row.names=FALSE)
  write.csv(data_z,"Glossina_marking-lab-Fie_ z.csv",row.names=FALSE)
  raw_data <- data[i_y,]

  ##basic test to see whether can identify dried samples
  X_all=data_X
  y_all.a=data_y
  y_all=ifelse(y_all.a=="irradiated-unmarked",0,1)
  raw_data_all=raw_data
  wvlenghts.range=350:2500
  wvlenghts=500:2400
  #raw_data_all=raw_data
  min.wv<-which(wvlenghts.range==min(wvlenghts))
```

```
 max.wv<-which(wvlenghts.range==max(wvlenghts))
 ii<-seq(1,length(y_all),1)
 ii<-which(raw_data_all[,"Sex"] == "Male")

#ii<-which(raw_data_all[,"Marking"] == "Field"  & raw_data_all[,"Sex"] == "Male")
 y2=y_all[ii]
 length(y2)
 table(y2)
 X=X_all[ii,min.wv:max.wv]
 raw_data=raw_data_all[ii,]
 set.seed(0145)
 obj_dgnostics<- fdaML_train(ll = list(X=X, y=y2, Z=NULL,
                         task="clas",
                         model="glm",
                         reduction="pls",
                         smooth_w=NULL, intercept=T,
                         lam_cv_type="n",
                         lam_vec=NULL,
                         reps=100, Q_len=NULL,
                         Q_opt=NULL, Q_vec=floor(seq(2, 50, 1)),
                         split_size=c(0.6,0.2,0.2), tau_Q_opt=0.05,
                         balanced=T, estimation_w=NULL,
                         bspline_dim=floor(1 * ncol(X)),
                         t_range=wvlenghts, verbose=T))
 fdaPlot(obj_dgnostics)
 obj_dgnostics$Q_opt
 obj_dgnostics$Q_vec

 ################### Spectre mean
 length(y2)
```

```
table(y2)
### plot average spectra
# gets spectra from class 0
x_0 <- X[which(y2==0),]
# get the average per column
x_0_avg <- colMeans(x_0)
## repeat for class 1
# gets spectra from class 1
x_1 <- X[which(y2==1),]
# get the average per column
x_1_avg <- colMeans(x_1)

average_to_plot <- data.frame("wavelengths"=wvlenghts,"irradiated-
unmarked"=x_0_avg,"Field"=x_1_avg)

#install.packages("ggplot2")
library(ggplot2)

none <- element_blank()
fig1A<-ggplot(average_to_plot,aes(x=wavelengths))+
  geom_line(aes(y=irradiated.unmarked ,col="sterile-unmarked flies"),size=1.9)+
  geom_line(aes(y=Field,col="wild-caught flies"),size=1.9)+
  theme_bw()+
  scale_colour_manual(values=c("blue","red"))+
  labs(y="Absorbance",x="Wavelengths (nm)",col="")+
  theme(legend.position = c(0.30, 0.8))+
  theme(panel.grid.major = none,panel.grid.minor = none)+
  theme(panel.background = none) + theme(panel.border = none) +
  theme(axis.text=element_text(size=14,colour="black"),axis.title=element_text(size=14))+
  theme(axis.line = element_line(colour = "black"))+
  theme(legend.text = element_text(colour = "black", size=16))+
```

```
  theme(legend.title = element_blank())+
  theme(legend.background = element_rect(fill="white"),legend.key = element_rect(fill = "white", color = NA))+
  theme(strip.text.x = element_text(size = 16))
fig1A
tiff(file= "figure spectre", res = 400, height = 5.0, width = 5, units = "in")
fig1A
dev.off()

###########################"Field"Versus"irradiated-marked"###################
rm(list = ls())
require(devtools)
setwd("C:/Users/Mouonniba B. SOME/Downloads/mlevcm")
#install.packages(c("rlang","caret", "dummies", "fda", "glmnet", "gplots", "penalized", "pls", "ROCR"))
#install.packages("mlevcm-master", type = "source", repos = NULL)
require("mlevcm")
##############################################
# read data
data<- read.csv("C:/Users/Mouonniba B. SOME/Desktop/Master Marx/Complement d'analyse_2024/Glossina Analysis_07052024.csv", header=T,sep = ";", na.strings = "NA")
data<- read.csv("C:/Users/Mouonniba B. SOME/Desktop/Master Marx/Complement d'analyse_2024/Glossina Analysis_07052024.csv", header=T,sep = ";", na.strings = "NA")
orig.id<-data[,"Glossina.ID1"]
data<-cbind(data,"Glossina.ID1")
target_marked = c("Field","irradiated-marked")

##following line removes all lines that are important which we have NAs for and can specifiy if you want a selection of rows (i.e. generation)
select <- (!is.na(data$Marking)) & (data[,"Marking"] %in% target_marked)#(!is.na(data$Age))
str(data)
unique_IDs <- unique(data[select,"Glossina.ID1"])
```

```
 ##following line removes all lines that are important which we have NAs for and can specifiy if you want a selection of rows (i.e. generation)

  idx <- list()

  for(i in unique_IDs){

    rng <- which(data[,"Glossina.ID1"] == i)

    aux <-  (!is.na(data[rng,"Marking"])) & (data[,"Marking"] %in% target_marked)# (!is.na(data[rng,"Age"]))

    idx[[i]] <- rng[aux]

  }

  n_replics <- unlist(lapply(idx, length))  # store how many repeated observations for each specimen



  # read predictor (X)

  dates<-        c("24August_21", "25August_21","29August_21","03Sept_21","05Sept_21","08Sept_21","10Sept_21","13Sept_21","14Sept_21", "15Sept_21","16Sept_21","17Sept_21","18Sept_21","19Sept_21", "21Sept_21","23Sept_21","24Sept_21","27Sept_21","28Sept_21","30Sept_21","02Oct_21","03Oct_21","05Oct_21","06Oct_21","08Oct_21","11Oct_21","13Oct_21","18Oct_21","19Oct_21","20Oct_21","21Oct_21","22Oct_21", "23Oct_21","28Oct_21", "20Feb_23","23Feb_23","26Feb_23", "01Mars_23","04Mars_23" )

  nx_dates_start <- c(  0 ,          79,        121,        223,        403,         435,      707,         739, 959,              1029 ,      1043,       1221,       1393,        1585,       1695,        1753,       1929, 2001,      2041,       2215,      2283,     2337,     2555,      2593,     2625,     2759,      2789, 2893,     2975,      3213,     3299,      3407,     3499,     3595,  6075,   7067,      7161,       7253, 7343)

  nx_dates_end   <- c(  78,          120,        222,        402,        434,       706,       738, 958,      1028,             1042,       1220,       1392,       1584,       1694,        1752,       1928, 2000,      2040,      2214,      2282,      2336,     2554,      2592,      2624,       2758,    2788, 2892,      2974,     3212,       3298,     3406,     3498,       3594,   3644,  6166,  7160,       7252, 7342,       7428 )

  nx_dates <- nx_dates_end - nx_dates_start + 1

  nx <- sum(nx_dates)# number of spectra files

  dx <- c()

  for(day in dates){

    dx_aux <- matrix(0, nx_dates[which(day==dates)], 2151);  kk <- 1
```

```
  for(k in nx_dates_start[which(day==dates)]:nx_dates_end[which(day==dates)]){
    if(day %in% c("24August_21",
"25August_21","29August_21","03Sept_21","05Sept_21","08Sept_21","10Sept_21","13Sept
_21","14Sept_21", "15Sept_21","16Sept_21","17Sept_21","18Sept_21","19Sept_21",
"21Sept_21","23Sept_21","24Sept_21","27Sept_21","28Sept_21","30Sept_21","02Oct_21","
03Oct_21","05Oct_21","06Oct_21","08Oct_21","11Oct_21","13Oct_21","18Oct_21","19Oct
_21","20Oct_21","21Oct_21","22Oct_21", "23Oct_21","28Oct_21")){
      f_name <- paste0("C:/Users/Mouonniba B. SOME/Desktop/Master Marx/Glossina data
Age specise2/Age-specise_",day,"_", formatC(k, width = 5,flag = 0),".txt")
    }
    else{
      f_name <- paste0("C:/Users/Mouonniba B. SOME/Desktop/Master Marx/Complement
d'analyse_2024/Glossina data analysis 2024/Age-specise_",day,"_", formatC(k, width = 5,flag
= 0),".txt")
}
    dx_aux[kk,] <- read.table(file = f_name, header=T, sep="\t")[,2]
    kk <- kk + 1
  }
  dx <- rbind(dx, dx_aux)
 }
 data_X <- dx[select,]
 # read response (y)
 i_y <- select #& ((1:nrow(data)) %in% unlist(sapply(idx, "[[", 1)))
 data_y <- data[i_y,"Marking"]
 data_z <- data[i_y,]

 # read response (y)

 setwd("C:/Users/Mouonniba B. SOME/Desktop/Master Marx/Complement
d'analyse_2024")
 #write your new files
 write.csv(data_X,"Glossina_marking-irradiated-marked-Fiel_ x.csv",row.names=FALSE)
 write.csv(data_y,"Glossina_marking-irradiated-marked-Fie_ y.csv",row.names=FALSE)
 write.csv(data_z,"Glossina_marking-irradiated-marked-Fie_ z.csv",row.names=FALSE)
```

```
  raw_data <- data[i_y,]

  ##basic test to see whether can identify dried samples
  X_all=data_X
  y_all.a=data_y
  y_all=ifelse(y_all.a=="irradiated-marked",0,1)
  raw_data_all=raw_data
  wvlenghts.range=350:2500
  wvlenghts=500:2400

#raw_data_all=raw_data
  min.wv<-which(wvlenghts.range==min(wvlenghts))
  max.wv<-which(wvlenghts.range==max(wvlenghts))
  ii<-seq(1,length(y_all),1)
  ii<-which(raw_data_all[,"Sex"] == "Male")

 #ii<-which(raw_data_all[,"Marking"] == "Field"  & raw_data_all[,"Sex"] == "Male")
  y2=y_all[ii]
  length(y2)
  table(y2)
  X=X_all[ii,min.wv:max.wv]
  raw_data=raw_data_all[ii,]
  set.seed(01345)
  obj_dgnostics<- fdaML_train(ll = list(X=X, y=y2, Z=NULL,
                                        task="clas",
                                        model="glm",
                                        reduction="pls",
                                        smooth_w=NULL, intercept=T,
                                        lam_cv_type="n",
                                        lam_vec=NULL,
```

```
                    reps=100, Q_len=NULL,
                    Q_opt=NULL, Q_vec=floor(seq(2, 50, 1)),
                    split_size=c(0.6,0.2,0.2), tau_Q_opt=0.05,
                    balanced=T, estimation_w=NULL,
                    bspline_dim=floor(1 * ncol(X)),
                    t_range=wvlenghts, verbose=T))

  fdaPlot(obj_dgnostics)
  obj_dgnostics$Q_opt
  obj_dgnostics$Q_vec

###########################"Field"versus"unirradiated_unmarked""###############
  rm(list = ls())
  require(devtools)
  setwd("C:/Users/Mouonniba B. SOME/Downloads/mlevcm")
  #install.packages(c("rlang","caret", "dummies", "fda", "glmnet", "gplots", "penalized", "pls", "ROCR"))
  #install.packages("mlevcm-master", type = "source", repos = NULL)
  require("mlevcm")
  ##############################################
  # read data
  data<- read.csv("C:/Users/Mouonniba B. SOME/Desktop/Master Marx/Complement d'analyse_2024/Glossina Analysis_07052024.csv", header=T,sep = ";", na.strings = "NA")
  orig.id<-data[,"Glossina.ID1"]
  data<-cbind(data,"Glossina.ID1")
  target_marked = c("Field","unirradiated_unmarked")
  select <- (!is.na(data$Marking)) & (data[,"Marking"] %in% target_marked)#(!is.na(data$Age))
  str(data)
  unique_IDs <- unique(data[select,"Glossina.ID1"])
  # indices of repetitions for each mosquito
  idx <- list()
```

```
  for(i in unique_IDs){
    rng <- which(data[,"Glossina.ID1"] == i)
    aux <-  (!is.na(data[rng,"Marking"])) & (data[,"Marking"] %in% target_marked)# (!is.na(data[rng,"Age"]))
    idx[[i]] <- rng[aux]
  }
  n_replics <- unlist(lapply(idx, length))  # store how many repeated observations for each specimen


  # read predictor (X)
  dates<-         c("24August_21", "25August_21","29August_21","03Sept_21","05Sept_21","08Sept_21","10Sept_21","13Sept_21","14Sept_21", "15Sept_21","16Sept_21","17Sept_21","18Sept_21","19Sept_21", "21Sept_21","23Sept_21","24Sept_21","27Sept_21","28Sept_21","30Sept_21","02Oct_21","03Oct_21","05Oct_21","06Oct_21","08Oct_21","11Oct_21","13Oct_21","18Oct_21","19Oct_21","20Oct_21","21Oct_21","22Oct_21", "23Oct_21","28Oct_21", "20Feb_23","23Feb_23","26Feb_23", "01Mars_23","04Mars_23" )
  nx_dates_start <- c(  0 ,          79,        121,        223,        403,         435,      707,        739, 959,              1029 ,      1043,       1221,       1393,        1585,        1695,        1753,       1929, 2001,       2041,        2215,       2283,     2337,      2555,       2593,      2625,     2759,       2789, 2893,     2975,       3213,     3299,      3407,     3499,     3595,  6075,   7067,       7161,        7253, 7343)
  nx_dates_end   <- c(  78,          120,        222,        402,        434,       706,       738, 958,      1028,              1042,       1220,       1392,       1584,        1694,        1752,       1928, 2000,       2040,       2214,       2282,       2336,     2554,       2592,      2624,        2758,    2788, 2892,       2974,     3212,        3298,     3406,     3498,        3594,   3644,  6166,  7160,       7252, 7342,       7428 )
  nx_dates <- nx_dates_end - nx_dates_start + 1
  nx <- sum(nx_dates)# number of spectra files
  dx <- c()
  for(day in dates){
    dx_aux <- matrix(0, nx_dates[which(day==dates)], 2151);  kk <- 1
    for(k in nx_dates_start[which(day==dates)]:nx_dates_end[which(day==dates)]){
      if(day %in% c("24August_21", "25August_21","29August_21","03Sept_21","05Sept_21","08Sept_21","10Sept_21","13Sept_21","14Sept_21", "15Sept_21","16Sept_21","17Sept_21","18Sept_21","19Sept_21", "21Sept_21","23Sept_21","24Sept_21","27Sept_21","28Sept_21","30Sept_21","02Oct_21","
```

```
03Oct_21","05Oct_21","06Oct_21","08Oct_21","11Oct_21","13Oct_21","18Oct_21","19Oct_21","20Oct_21","21Oct_21","22Oct_21", "23Oct_21","28Oct_21")){
        f_name <- paste0("C:/Users/Mouonniba B. SOME/Desktop/Master Marx/Glossina data Age specise2/Age-specise_",day,"_", formatC(k, width = 5,flag = 0),".txt")
      }
      else{
        f_name <- paste0("C:/Users/Mouonniba B. SOME/Desktop/Master Marx/Complement d'analyse_2024/Glossina data analysis 2024/Age-specise_",day,"_", formatC(k, width = 5,flag = 0),".txt")
      }
      dx_aux[kk,] <- read.table(file = f_name, header=T, sep="\t")[,2]
      kk <- kk + 1
    }
    dx <- rbind(dx, dx_aux)
  }
  data_X <- dx[select,]
  # read response (y)
  i_y <- select #& ((1:nrow(data)) %in% unlist(sapply(idx, "[[", 1)))
  data_y <- data[i_y,"Marking"]
  data_z <- data[i_y,]
  setwd("C:/Users/Mouonniba B. SOME/Desktop/Master Marx/Complement d'analyse_2024")
  #write your new files
  write.csv(data_X,"Glossina_marking-unirradiated_unmarked-Fiel_x.csv",row.names=FALSE)
  write.csv(data_y,"Glossina_marking-unirradiated_unmarked-Fie_y.csv",row.names=FALSE)
  write.csv(data_z,"Glossina_marking-unirradiated_unmarked-Fie_z.csv",row.names=FALSE)
  raw_data <- data[i_y,]


  ##basic test to see whether can identify dried samples
  X_all=data_X
```

```
y_all.a=data_y
y_all=ifelse(y_all.a=="unirradiated_unmarked",0,1)
raw_data_all=raw_data
wvlenghts.range=350:2500
wvlenghts=500:2400
#raw_data_all=raw_data
min.wv<-which(wvlenghts.range==min(wvlenghts))
max.wv<-which(wvlenghts.range==max(wvlenghts))
ii<-seq(1,length(y_all),1)
ii<-which(raw_data_all[,"Sex"] == "Male")
#ii<-which(raw_data_all[,"Marking"] == "Field"  & raw_data_all[,"Sex"] == "Male")
y2=y_all[ii]
length(y2)
table(y2)
X=X_all[ii,min.wv:max.wv]
raw_data=raw_data_all[ii,]
set.seed(02350)
obj_dgnostics<- fdaML_train(ll = list(X=X, y=y2, Z=NULL,
                          task="clas",
                          model="glm",
                          reduction="pls",
                          smooth_w=NULL, intercept=T,
                          lam_cv_type="n",
                          lam_vec=NULL,
                          reps=100, Q_len=NULL,
                          Q_opt=NULL, Q_vec=floor(seq(2, 50, 1)),
                          split_size=c(0.6,0.2,0.2), tau_Q_opt=0.05,
                          balanced=T, estimation_w=NULL,
                          bspline_dim=floor(1 * ncol(X)),
                          t_range=wvlenghts, verbose=T))
```

```
  fdaPlot(obj_dgnostics)
  obj_dgnostics$Q_opt
  obj_dgnostics$Q_vec

 ##########################"unirradiated_unmarked","irradiated-unmarked""########
  rm(list = ls())
  require(devtools)
  setwd("C:/Users/Mouonniba B. SOME/Downloads/mlevcm")
  #install.packages(c("rlang","caret", "dummies", "fda", "glmnet", "gplots", "penalized", "pls",
"ROCR"))
  #install.packages("mlevcm-master", type = "source", repos = NULL)
  require("mlevcm")
  ################################################

   # read data
  data<- read.csv("C:/Users/Mouonniba B. SOME/Desktop/Master Marx/Complement
d'analyse_2024/Glossina Analysis_07052024.csv", header=T,sep = ";", na.strings = "NA")
  orig.id<-data[,"Glossina.ID1"]
  data<-cbind(data,"Glossina.ID1")
  target_marked = c("unirradiated_unmarked","irradiated-unmarked")
  select <- (!is.na(data$Marking)) & (data[,"Marking"] %in%
target_marked)#(!is.na(data$Age))
  str(data)
  unique_IDs <- unique(data[select,"Glossina.ID1"])
  ##following line removes all lines that are important which we have NAs for and can
specifiy if you want a selection of rows (i.e. generation)
  idx <- list()
  for(i in unique_IDs){
    rng <- which(data[,"Glossina.ID1"] == i)
    aux <-  (!is.na(data[rng,"Marking"])) & (data[,"Marking"] %in% target_marked)#
(!is.na(data[rng,"Age"]))
```

```
    idx[[i]] <- rng[aux]
  }
  n_replics <- unlist(lapply(idx, length))  # store how many repeated observations for each
specimen


  # read predictor (X)
  dates<-         c("24August_21",
"25August_21","29August_21","03Sept_21","05Sept_21","08Sept_21","10Sept_21","13Sept
_21","14Sept_21", "15Sept_21","16Sept_21","17Sept_21","18Sept_21","19Sept_21",
"21Sept_21","23Sept_21","24Sept_21","27Sept_21","28Sept_21","30Sept_21","02Oct_21","
03Oct_21","05Oct_21","06Oct_21","08Oct_21","11Oct_21","13Oct_21","18Oct_21","19Oct
_21","20Oct_21","21Oct_21","22Oct_21", "23Oct_21","28Oct_21",
"20Feb_23","23Feb_23","26Feb_23", "01Mars_23","04Mars_23" )
  nx_dates_start <- c(  0 ,          79,        121,        223,       403,        435,      707,        739,
959,               1029 ,      1043,       1221,       1393,       1585,       1695,       1753,       1929,
2001,       2041,        2215,       2283,     2337,      2555,       2593,      2625,     2759,       2789,
2893,     2975,       3213,      3299,      3407,      3499,      3595,  6075,   7067,       7161,        7253,
7343)
  nx_dates_end   <- c(  78,            120,        222,        402,        434,       706,       738,
958,      1028,              1042,       1220,       1392,       1584,       1694,       1752,       1928,
2000,       2040,       2214,       2282,       2336,     2554,       2592,      2624,       2758,    2788,
2892,      2974,     3212,        3298,     3406,      3498,        3594,   3644,  6166,  7160,       7252,
7342,       7428 )
  nx_dates <- nx_dates_end - nx_dates_start + 1
  nx <- sum(nx_dates)# number of spectra files
  dx <- c()
  for(day in dates){
    dx_aux <- matrix(0, nx_dates[which(day==dates)], 2151);  kk <- 1
    for(k in nx_dates_start[which(day==dates)]:nx_dates_end[which(day==dates)]){
      if(day %in% c("24August_21",
"25August_21","29August_21","03Sept_21","05Sept_21","08Sept_21","10Sept_21","13Sept
_21","14Sept_21", "15Sept_21","16Sept_21","17Sept_21","18Sept_21","19Sept_21",
"21Sept_21","23Sept_21","24Sept_21","27Sept_21","28Sept_21","30Sept_21","02Oct_21","
03Oct_21","05Oct_21","06Oct_21","08Oct_21","11Oct_21","13Oct_21","18Oct_21","19Oct
_21","20Oct_21","21Oct_21","22Oct_21", "23Oct_21","28Oct_21")){
        f_name <- paste0("C:/Users/Mouonniba B. SOME/Desktop/Master Marx/Glossina data
Age specise2/Age-specise_",day,"_", formatC(k, width = 5,flag = 0),".txt")
      }
```

```
    else{
      f_name <- paste0("C:/Users/Mouonniba B. SOME/Desktop/Master Marx/Complement d'analyse_2024/Glossina data analysis 2024/Age-specise_",day,"_", formatC(k, width = 5,flag = 0),".txt")
    }
    dx_aux[kk,] <- read.table(file = f_name, header=T, sep="\t")[,2]
    kk <- kk + 1
   }
  dx <- rbind(dx, dx_aux)
 }
 data_X <- dx[select,]
 # read response (y)
 i_y <- select #& ((1:nrow(data)) %in% unlist(sapply(idx, "[[", 1)))
 data_y <- data[i_y,"Marking"]
 data_z <- data[i_y,]

 setwd("C:/Users/Mouonniba B. SOME/Desktop/Master Marx/Complement d'analyse_2024")
 #write your new files
 write.csv(data_X,"Glossina_marking-Irradiation effect_ x.csv",row.names=FALSE)
 write.csv(data_y,"Glossina_marking-Irradiation effect_ y.csv",row.names=FALSE)
 write.csv(data_z,"Glossina_marking-Irradiation effect_ z.csv",row.names=FALSE)
 raw_data <- data[i_y,]

 ##basic test to see whether can identify dried samples
 X_all=data_X
 y_all.a=data_y
 y_all=ifelse(y_all.a=="unirradiated_unmarked",0,1)
 raw_data_all=raw_data
 wvlenghts.range=350:2500
 wvlenghts=500:2400
 #raw_data_all=raw_data
```

```
min.wv<-which(wvlenghts.range==min(wvlenghts))
max.wv<-which(wvlenghts.range==max(wvlenghts))
ii<-seq(1,length(y_all),1)
ii<-which(raw_data_all[,"Sex"] == "Male")
#ii<-which(raw_data_all[,"Marking"] == "Field"  & raw_data_all[,"Sex"] == "Male")
y2=y_all[ii]
length(y2)
table(y2)
X=X_all[ii,min.wv:max.wv]
raw_data=raw_data_all[ii,]
set.seed(140051)
obj_dgnostics<- fdaML_train(ll = list(X=X, y=y2, Z=NULL,
                              task="clas",
                              model="glm",
                              reduction="pls",
                              smooth_w=NULL, intercept=T,
                              lam_cv_type="n",
                              lam_vec=NULL,
                              reps=100, Q_len=NULL,
                              Q_opt=NULL, Q_vec=floor(seq(2, 50, 1)),
                              split_size=c(0.6,0.2,0.2), tau_Q_opt=0.05,
                              balanced=T, estimation_w=NULL,
                              bspline_dim=floor(1 * ncol(X)),
                              t_range=wvlenghts, verbose=T))

fdaPlot(obj_dgnostics)
obj_dgnostics$Q_opt
obj_dgnostics$Q_vec

##########################"irradiated-unmarked"versus"irradiated-marked"#########
```

```
  rm(list = ls())

  require(devtools)

  setwd("C:/Users/Mouonniba B. SOME/Downloads/mlevcm")

  #install.packages(c("rlang","caret", "dummies", "fda", "glmnet", "gplots", "penalized", "pls",
"ROCR"))

  #install.packages("mlevcm-master", type = "source", repos = NULL)

  require("mlevcm")

  ###############################################


  # read data

  data<- read.csv("C:/Users/Mouonniba B. SOME/Desktop/Master Marx/Complement
d'analyse_2024/Glossina Analysis_07052024.csv", header=T,sep = ";", na.strings = "NA")

  orig.id<-data[,"Glossina.ID1"]

  data<-cbind(data,"Glossina.ID1")

  target_marked = c("irradiated-unmarked","irradiated-marked")

  select <- (!is.na(data$Marking)) & (data[,"Marking"] %in%
target_marked)#(!is.na(data$Age))

  str(data)

  unique_IDs <- unique(data[select,"Glossina.ID1"])

  ##following line removes all lines that are important which we have NAs for and can
specifiy if you want a selection of rows (i.e. generation)

  idx <- list()

  for(i in unique_IDs){

    rng <- which(data[,"Glossina.ID1"] == i)

    aux <-  (!is.na(data[rng,"Marking"])) & (data[,"Marking"] %in% target_marked)#
(!is.na(data[rng,"Age"]))

    idx[[i]] <- rng[aux]

  }

  n_replics <- unlist(lapply(idx, length))  # store how many repeated observations for each
specimen


  # read predictor (X)
```

```
dates<-         c("24August_21",
"25August_21","29August_21","03Sept_21","05Sept_21","08Sept_21","10Sept_21","13Sept
_21","14Sept_21", "15Sept_21","16Sept_21","17Sept_21","18Sept_21","19Sept_21",
"21Sept_21","23Sept_21","24Sept_21","27Sept_21","28Sept_21","30Sept_21","02Oct_21","
03Oct_21","05Oct_21","06Oct_21","08Oct_21","11Oct_21","13Oct_21","18Oct_21","19Oct
_21","20Oct_21","21Oct_21","22Oct_21", "23Oct_21","28Oct_21",
"20Feb_23","23Feb_23","26Feb_23", "01Mars_23","04Mars_23" )

nx_dates_start <- c(  0 ,          79,        121,        223,        403,         435,      707,         739,
959,              1029 ,      1043,       1221,       1393,        1585,        1695,        1753,       1929,
2001,       2041,        2215,       2283,      2337,       2555,        2593,       2625,      2759,       2789,
2893,     2975,       3213,      3299,       3407,      3499,      3595,  6075,   7067,       7161,        7253,
7343)

nx_dates_end   <- c(  78,           120,        222,        402,        434,       706,       738,
958,      1028,             1042,       1220,       1392,       1584,       1694,        1752,        1928,
2000,      2040,       2214,       2282,       2336,      2554,       2592,      2624,        2758,    2788,
2892,      2974,     3212,        3298,     3406,     3498,        3594,   3644,  6166,  7160,       7252,
7342,       7428 )

nx_dates <- nx_dates_end - nx_dates_start + 1

nx <- sum(nx_dates)# number of spectra files

dx <- c()

for(day in dates){

  dx_aux <- matrix(0, nx_dates[which(day==dates)], 2151);  kk <- 1

  for(k in nx_dates_start[which(day==dates)]:nx_dates_end[which(day==dates)]){

    if(day %in% c("24August_21",
"25August_21","29August_21","03Sept_21","05Sept_21","08Sept_21","10Sept_21","13Sept
_21","14Sept_21", "15Sept_21","16Sept_21","17Sept_21","18Sept_21","19Sept_21",
"21Sept_21","23Sept_21","24Sept_21","27Sept_21","28Sept_21","30Sept_21","02Oct_21","
03Oct_21","05Oct_21","06Oct_21","08Oct_21","11Oct_21","13Oct_21","18Oct_21","19Oct
_21","20Oct_21","21Oct_21","22Oct_21", "23Oct_21","28Oct_21")){

      f_name <- paste0("C:/Users/Mouonniba B. SOME/Desktop/Master Marx/Glossina data
Age specise2/Age-specise_",day,"_", formatC(k, width = 5,flag = 0),".txt")

    }

    else{

      f_name <- paste0("C:/Users/Mouonniba B. SOME/Desktop/Master Marx/Complement
d'analyse_2024/Glossina data analysis 2024/Age-specise_",day,"_", formatC(k, width = 5,flag
= 0),".txt")

    }

    dx_aux[kk,] <- read.table(file = f_name, header=T, sep="\t")[,2]
```

```
    kk <- kk + 1
   }
   dx <- rbind(dx, dx_aux)
  }
  data_X <- dx[select,]
  # read response (y)
  i_y <- select #& ((1:nrow(data)) %in% unlist(sapply(idx, "[[", 1)))
  data_y <- data[i_y,"Marking"]
  data_z <- data[i_y,]

  setwd("C:/Users/Mouonniba B. SOME/Desktop/Master Marx/Complement
d'analyse_2024")
  #write your new files
  write.csv(data_X,"Glossina_marking effect_ x.csv",row.names=FALSE)
  write.csv(data_y,"Glossina_marking effect_ y.csv",row.names=FALSE)
  write.csv(data_z,"Glossina_marking effect_ z.csv",row.names=FALSE)
  raw_data <- data[i_y,]

  ##basic test to see whether can identify dried samples
  X_all=data_X
  y_all.a=data_y
  y_all=ifelse(y_all.a=="irradiated-unmarked",0,1)
  raw_data_all=raw_data
  wvlenghts.range=350:2500
  wvlenghts=500:2400

  #raw_data_all=raw_data
  min.wv<-which(wvlenghts.range==min(wvlenghts))
  max.wv<-which(wvlenghts.range==max(wvlenghts))
  ii<-seq(1,length(y_all),1)
  ii<-which(raw_data_all[,"Sex"] == "Male")
```

```
#ii<-which(raw_data_all[,"Marking"] == "Field"  & raw_data_all[,"Sex"] == "Male")
y2=y_all[ii]
length(y2)
table(y2)
X=X_all[ii,min.wv:max.wv]
raw_data=raw_data_all[ii,]
set.seed(0055)
obj_dgnostics<- fdaML_train(ll = list(X=X, y=y2, Z=NULL,
                          task="clas",
                          model="glm",
                          reduction="pls",
                          smooth_w=NULL, intercept=T,
                          lam_cv_type="n",
                          lam_vec=NULL,
                          reps=100, Q_len=NULL,
                          Q_opt=NULL, Q_vec=floor(seq(2, 50, 1)),
                          split_size=c(0.6,0.2,0.2), tau_Q_opt=0.05,
                          balanced=T, estimation_w=NULL,
                          bspline_dim=floor(1 * ncol(X)),
                          t_range=wvlenghts, verbose=T))

fdaPlot(obj_dgnostics)
obj_dgnostics$Q_opt
obj_dgnostics$Q_vec
################### Graphs adjustments ################################
obj <- obj_dgnostics
layout(matrix(c(1,3,2,3), 2, 2, byrow = TRUE),heights=c(2,2),widths = c(2,2))
#par(mfrow=c(1,3), mai = c(1, 0.1, 0.1, 0.1))
par(mar=2+c(1.95,1.95,1.95,1.95),mai = c(0.5, 0.7, 0.1, 0.4))
```

```
  #tiff(file="Figure_marking",res = 400, height = 5, width = 5, units = "in")
  ### Q cross-validation
  #pdf(file = "C:/Users/Mouonniba B. SOME/Desktop/Master Marx/Complement d'analyse_2024/fig_diagnostics_Qcv.pdf", height=5, width=5, pointsize=12)
  #{
  #avg_auc <- round(colMeans(obj$perf_cv)[obj$Qvec==obj$Qopt],2)
  #matplot(obj$Q_vec, t(obj$perf_cv), type="l", lty=1, col="grey", xlab="components (Q)", ylab="AUC", ylim=c(0,1))
  #lines(obj$Q_vec, colMeans(obj$perf_cv), lwd=2)
  #abline(v = c(obj$Q_opt), lty=2)
  #legend("bottomright", c("Q-AUC curves", "mean Q-AUC curve", paste0("optimal Q = ", obj$Q_opt)), lty=c(1,1,2), lwd=c(1,2,1), col=c('grey','black','black'), seg.len=1.2, bty="n", cex=0.9)
  #legend("topleft", expression(bold(a)), bty="n")}
  #dev.off()


  ### cutoffs
  #pdf(file = "C:/1.Work/Pedro handover/figures/fig_diagnostics_cutoffs.pdf", height=5, width=5, pointsize=12)
  {
    pred <- prediction(obj$ROC_to_plot_binom$pred_test, obj$ROC_to_plot_binom$labels)
    perf <- performance(pred, "tpr", "fpr")
    # stats
    err_perf <- performance(pred, measure = "err")
    err_ind = sapply(1:obj$reps, function(z){ which.min(err_perf@y.values[[z]]) })
    if(any(err_ind == 1)){   # get rid of infinities in 'err_perf@x.values)'
      tt <- (1:obj$reps)[err_ind == 1]; for(l in 1:length(tt)){ if( is.infinite(slot(err_perf, "x.values")[[tt[l]]][1]) ){ err_ind[tt[l]] <- 2 }}
    }
    err_val = sapply(1:obj$reps, function(z){ err_perf@y.values[[z]][err_ind[z]] })
    err_cut = sapply(1:obj$reps, function(z){ err_perf@x.values[[z]][err_ind[z]] })
    avg_err <- mean(err_val)
    avg_err_cut <- mean(err_cut)
```

```
    error_per_rep <- sapply(1:obj$reps, function(qq){ mean((obj$y_testpred_opt[,qq] > avg_err_cut) != obj$y[obj$id_test[[qq]]]) })

    avg_err_avgcut <- mean(error_per_rep)

    stdev_err_avgcut <- sd(error_per_rep)

    #err_colcode <- ifelse( all(avg_err_avgcut < c(0.5, mean(obj$y==1), mean(obj$y==0))), "green", ifelse(all(avg_err_avgcut < 0.5), "orange", "red") )

    #d_allcurves <- data.frame(x = unlist(err_perf@x.values), y = unlist(err_perf@y.values)); d_allcurves <- d_allcurves[order(d_allcurves$x),]

    # plot

    #plot(err_perf, lty=1, col="grey", xlim=c(0,1), ylim=c(0,1), xlab="cutoff (probability)", ylab="error rate")   # cutoff-error curve for each randomisation (1:obj$reps)

    #lines(d_allcurves$x[!is.infinite(d_allcurves$x)], predict(loess(d_allcurves$y[!is.infinite(d_allcurves$x)] ~ d_allcurves$x[!is.infinite(d_allcurves$x)])), lwd=2)

    #points(err_cut,err_val)                                    # optimal (cutoff,error) point for each randomisation (1:obj$reps)

    #abline(v = avg_err_cut, lty=2)                                    # average cutoff

    #points(avg_err_cut, avg_err, pch=19, col="black", cex=1.3)             # avg error (w/ curve-specific cutoff)

    #points(avg_err_cut, avg_err_avgcut, pch=19, col=err_colcode, cex=1.3)   # avg error (w/ avg cutoff)

    #legend("topright", c("cutoff-error curves", "mean cutoff-error curve", "avg cutoff", paste0("avg error (w/ curve-specific cutoff) = ", round(avg_err,2)), paste0("avg error (w/ avg cutoff) = ", round(avg_err_avgcut,2)), paste0("freq minority class = ", round(length(obj$y[obj$y == names(table(obj$y))[which.min(table(obj$y))]]) / length(obj$y),2))), lty=c(1,1,2,NA,NA,NA), pch=c(NA,NA,NA,19,19,NA), col=c("grey","black","black","black",err_colcode,NA), seg.len=1.2,  bty="n", cex=0.9)

    #legend("topleft", expression(bold(b)), bty="n")

  }

  #dev.off()




  ### AUcs

  #pdf(file = "C:/1.Work/Pedro handover/figures/fig_diagnostics_auc.pdf", height=5, width=5, pointsize=12)

  {
```

```
avg_auc <- mean(obj$AUC_opt[,"test"]) #
round(colMeans(obj$perf_cv)[obj$Qvec==obj$Qopt],2) #

pred <- prediction(obj$ROC_to_plot_binom$pred_test, obj$ROC_to_plot_binom$labels)

perf <- performance(pred, "tpr", "fpr")

dd <- data.frame(x = unlist(perf@x.values), y = unlist(perf@y.values)); dd <-
dd[order(dd[,1]),]

xvals <- seq(0, 1, by=0.1);   lx <- length(xvals)

intervals <- matrix(c(xvals-0.05, xvals+0.05), lx, 2);   idx <- matrix(0, lx, 2)

for(k in 1:nrow(intervals)){ idx[k,] <- range(which(dd$x >= intervals[k,1] & dd$x <
intervals[k,2])) }

forget <- which(is.infinite(idx[,1]) * is.infinite(idx[,2]) == 1)

idx[forget,] <- 0

ofs_x <- 0.032

percentile_values <- c(0.05, 0.15, 0.25, 0.5, 0.75, 0.85, 0.95)

percentiles <- matrix(0, length(percentile_values), lx);   x_vec <- matrix(0, 2, lx);   means
<- stdev <- rep(0,lx)

for(u in 1:lx){

  percentiles[,u] <- quantile(dd$y[idx[u,1]:idx[u,2]], probs=percentile_values)

  x_vec[,u]       <- c(xvals[u] - ofs_x, xvals[u] + ofs_x)

  means[u]        <- mean(dd$y[idx[u,1]:idx[u,2]])

  stdev[u]        <- sd(dd$y[idx[u,1]:idx[u,2]])

}

# plot

plot(perf, col="grey", lty=1, ylim=c(0,1), xlab="false positive rate", ylab="true positive
rate")

for(u in 1:lx){

  # BOXES

  rect(x_vec[1,u], percentiles[3,u], x_vec[2,u], percentiles[5,u], col="white") # fill boxes

  segments(x_vec[1,u], percentiles[4,u], x_vec[2,u], percentiles[4,u], lty=1, lwd=3, lend=1)

  # WHISKERS

  segments(xvals[u], percentiles[5,u], xvals[u], percentiles[7,u], lty=1, lwd=1, lend=1)

  segments(xvals[u], percentiles[3,u], xvals[u], percentiles[1,u], lty=1, lwd=1, lend=1)
```

```
      # WHISKER ENDS
      segments(xvals[u]-ofs_x/2, percentiles[6,u], xvals[u]+ofs_x/2, percentiles[6,u], lty=1, lwd=1, lend=1)
      segments(xvals[u]-ofs_x/2, percentiles[2,u], xvals[u]+ofs_x/2, percentiles[2,u], lty=1, lwd=1, lend=1)
      segments(xvals[u]-ofs_x/4, percentiles[7,u], xvals[u]+ofs_x/4, percentiles[7,u], lty=1, lwd=1, lend=1)
      segments(xvals[u]-ofs_x/4, percentiles[1,u], xvals[u]+ofs_x/4, percentiles[1,u], lty=1, lwd=1, lend=1)
    }
    if(length(forget) == 0){
      lines(xvals, predict(loess(means ~ xvals)), lty=1, lwd=2)
    }else{
      lines(xvals[-forget], predict(loess(means ~ xvals)), lty=1, lwd=2)
    }
    # OR just confidence intervals instead of boxplots
    #lines(xvals, predict(loess(extremes[1,] ~ xvals)), lty=2, lwd=1); lines(xvals, predict(loess(extremes[2,] ~ xvals)), lty=2, lwd=1)
    text(x = xvals, y = percentiles[5,]-0.025, labels = sapply(1:length(xvals), function(aa){ paste0(strsplit(as.character(round(stdev,2)), split="")[[aa]][2:4], collapse='') }), col="black", cex=0.75)
    labls <- c(paste0("avg AUC = ", round(avg_auc,2)), "ROC curves", "mean ROC curve", "standard deviation")
    legend("bottomright", legend=labls, lty=c(NA,1,1,NA), lwd=c(NA,1,2,NA), col=c(NA,'grey','black','black'), bty="n", seg.len=1.2, cex=1.2)
    text(x=0.59, y=0.02, labels="", cex=1.2)
    legend("topleft", expression(bold(a)), bty="n")
  }
  #dev.off()



  ### coefficient function
  #pdf(file = "C:/1.Work/Pedro handover/figures/fig_diagnostics_coef.pdf", height=5, width=5, pointsize=12)
```

```
  {
    matplot(obj$t_range_mod, obj$beta, type="l", lty=1, col=alpha("gray",0.6), xlab="wavelength", ylab="coefficient function")
    lines(obj$t_range_mod, rowMeans(obj$beta), type="l", lwd=2 , col="black")
    legend("bottomright", c("coefficient functions", "mean coefficient function"), lty=c(1,1), lwd=c(1,2), col=c('grey','black'), seg.len=1.2, bty="n", cex=1.2)
    legend("topleft", expression(bold(b)), bty="n")
  }
  #dev.off()


  tt=1
  ### densities and confusion matrix
  #pdf(file = "C:/1.Work/Pedro handover/figures/fig_diagnostics_densities2.pdf", height=5, width=10, pointsize=12)
  {
    oldparams <- par()
    hist_range <- c(-300,300)
    id1 <- c(sapply(1:obj$reps, function(z){ obj$y[obj$id_test[[z]]]==1 }))
    d <- data.frame(label = as.factor(id1+0), val_linpred = c(obj$linear_predictor)); d$val_prob <- 1/(1+exp(-d$val_linpred))
    nbins <- 40;   col0 <- rgb(0,1,0,0.5);   col1 <- rgb(0,0,1,0.55)
    optcut <- -log((1-avg_err_cut)/avg_err_cut) # obtained by solving 'P = 1 / (1 + exp(-L))' for the linear predictor 'L'
    dd <- d$val_linpred
    n1 <- hist_range[1];       n2 <- hist_range[2];      wd <- n2-n1;  perc <- abs(range(hist_range)-optcut)/wd
    brks <- c(seq(n1, optcut, len=perc[1]*nbins), seq(optcut, n2, len=perc[2]*nbins+1)[-1])
    confu <- confusionMatrix(data=as.factor(as.numeric(c(obj$y_testpred_opt) > avg_err_cut)), reference = as.factor(obj$y[unlist(obj$id_test)]))$table
    dc <- list(tnr = confu[1,1] / sum(confu[,1]),
               tpr = confu[2,2] / sum(confu[,2]),
               fnr = confu[1,2] / sum(confu[,2]),
               fpr = confu[2,1] / sum(confu[,1]))
```

```
    hist(dd[d$label==0 & dd > hist_range[1] & dd < hist_range[2]], col=col0, freq=F, breaks=brks, border="white", xlab=ifelse(tt=="prob","probability of class 1","linear predictor"), ylab="density", main="")

    hist(dd[d$label==1 & dd > hist_range[1] & dd < hist_range[2]], col=col1, freq=F, breaks=brks, border="white", add=T)

    abline(v=optcut, lwd=2)

    legend("topleft", c(paste0("avg. error=",round(avg_err_avgcut,2)), "optimal cutoff", expression(bold("")), "Sterile-unmark : 0", "Sterile-mark : 1", ""), lty=c(NA,1,NA,1,1,NA), lwd=c(NA,2,NA,NA,NA,NA), pch=c(NA,NA,NA,15,15,NA), col=c(NA,"black",NA,col0,col1,NA), seg.len=1.2, bty="n", cex=1.2)

    par(fig = c(0.78, 1, 0.6, 0.95), new=TRUE, mar=2+c(0,0,0,0))

    #par(fig = c(0.7, 1, 0.6, 0.9), new=TRUE, mar=2+c(0,0,0,0))

    confusion_plot(d=dc, fam=obj$family)

    options(showWarnCalls = F)

    par(oldparams)

    options(showWarnCalls = T)

    legend("bottomleft", expression(bold(c)), bty="n")

  }

  mtext(at=c(900),"December (fresh state) coluzzii",line=0.2,cex = 1.2)

  dev.off()
```
